# Chemotherapy for advanced pulmonary sarcomatoid carcinoma: a population-based propensity score matching study

**DOI:** 10.1186/s12890-023-02541-1

**Published:** 2023-07-15

**Authors:** Feng Wang, Xiangyang Yu, Yi Han, Changfan Gong, Dongjie Yan, Lei Yang, Jie Li, Shuku Liu

**Affiliations:** 1 Beijing Chest Hospital, Capital Medical University, Beijing, 101125 China; 2 Beijing Tuberculosis and Thoracic Tumor Research Institute, Beijing, 101125 China; 3 Department of Thoracic Surgery, National Cancer Center/National Clinical Research Center for Cancer, Cancer Hospital & Shenzhen Hospital, Chinese Academy of Medical Sciences and Peking Union Medical College, Shenzhen, China

**Keywords:** Pulmonary sarcomatoid carcinoma (PSC), Surveillance, Epidemiology, and End Results (SEER) database, Propensity score matching (PSM)

## Abstract

**Objectives:**

Pulmonary sarcomatoid carcinoma (PSC) is a rare histological type of non-small cell lung cancer (NSCLC). There are no specific treatment guidelines for PSC. For advanced PSC (stage II-IV), the role of chemotherapy is still controversial. The purpose of this study was to investigate the effect of chemotherapy on the prognosis of advanced PSC.

**Methods:**

A total of 960 patients with advanced PSC from the Surveillance, Epidemiology, and End Results (SEER) database between 2010 and 2019 were enrolled in this study. To investigate the prognostic factors, the Cox proportional hazard regression model was conducted. A total of 642 cases were obtained after propensity score matching (PSM). The Kaplan‒Meier method was applied to compare overall survival (OS) and cancer-specific survival (CSS).

**Results:**

For all 960 cases included in this study, the Cox proportional hazard model was applied for prognostic analysis. Univariate and multivariate analyses showed that stage, T stage, N stage, M stage, surgery, and chemotherapy were prognostic factors for OS and CSS (*P* < 0.05). A total of 642 cases were obtained after PSM, with no significant difference between the two groups for all variables. Kaplan‒Meier curves indicated that for OS and CSS, the prognosis was significantly better in the chemotherapy group than in the no-chemotherapy group.

**Conclusions:**

For advanced PSC, chemotherapy can significantly improve the OS and CSS of patients. Chemotherapy should be an important part of PSC treatment.

## Introduction

Pulmonary sarcomatoid carcinoma (PSC) is a rare pathologic subtype of non-small cell lung cancer (NSCLC) that accounts for approximately 0.1–0.4% of all lung malignancies [1, 2]. According to the 2021 World Health Organization tumor classification system, PSC includes the following five pathologic types: spindle cell carcinoma, pleomorphic carcinoma, carcinosarcoma, giant cell carcinoma, and pulmonary blastoma [3]. Older male patients make up the majority of PSC patients, and most patients have a history of smoking [4]. Patients with PSC do not have a characteristic clinical manifestation, and typical chest CT features are large solid masses located in the periphery of the lungs [4–8]. Due to the high aggressiveness of PSC, its prognosis is usually poor, with a 5-year overall survival rate of approximately 12.6–34.6% [4, 9–11]. For early-stage PSC, surgery is the best treatment option. For advanced PSC, there is still no standard treatment.

Since there are no treatment guidelines for PSC, chemotherapy for PSC currently follows the chemotherapy regimen for NSCLC. However, there is still controversy about the value of chemotherapy in advanced PSC [12–19], so the purpose of this study was to investigate the impact of chemotherapy on the prognosis of advanced PSC using data from the Surveillance, Epidemiology, and End Results (SEER) database.

## Methods

### Data source

**Fig. 1:**
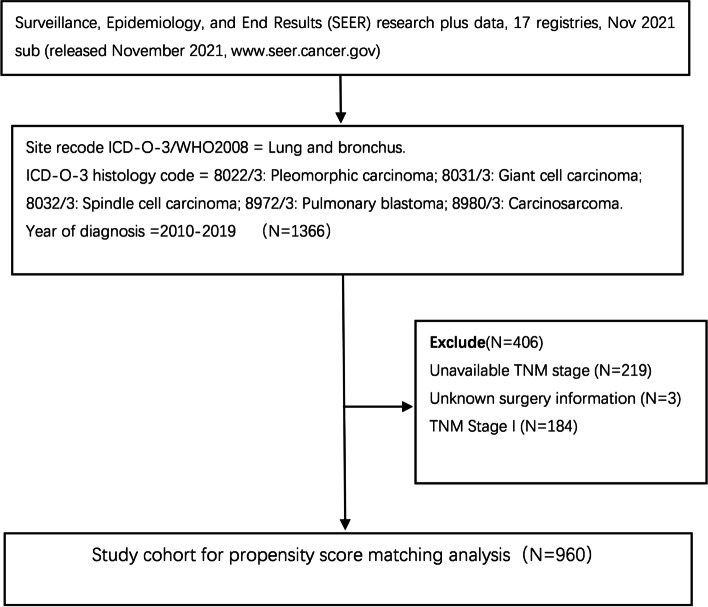
Flow chart of the cases selection

This study is a retrospective study based on the SEER database. The detailed database information is Surveillance, Epidemiology, and End Results (SEER) Program (www.seer.cancer.gov) SEER*Stat Database: Incidence—SEER Research Plus Data, 17 Registries, Nov 2021 Sub (1975–2020)—Linked To County Attributes—Time Dependent (1990–2020) Income/Rurality, 1969–2020 Counties, National Cancer Institute, DCCPS (Division of Cancer Control and Population Sciences), Surveillance Research Program, released April 2022, based on the November 2021 submission.Surveillance Research Program, National Cancer Institute SEER*Stat software (seer.cancer.gov/seerstat) version < 8.4.0 > was installed to extract data.The SEER research data agreement was signed, and access to the database was obtained prior to data acquisition. The criteria for case selection were as follows: (I) diagnosis from 2010 to 2019, (II) location as lung and bronchus, (III) International Classification of Disease for Oncology, 3rd edition (ICD-O-3) code and histological information as 8022/3: pleomorphic carcinoma; 8031/3: giant cell carcinoma; 8032/3: spindle cell carcinoma; 8972/3: pulmonary blastoma; 8980/3: carcinosarcoma. Exclusion criteria were as follows: (I) no TNM staging information, (II) no surgery information, and (III) TNM stage I. A total of 960 cases of advanced PSC diagnosed between 2010 and 2019 were eventually included in this study (Fig. 1).

### Outcome

The variables included in this study were age, sex, marital status, race, laterality, histology, TNM stage, T stage, N stage, M stage, surgery, radiotherapy, and chemotherapy. Patient deaths from all causes were regarded as uncensored cases for the overall survival (OS) analysis, while the cancer-specific survival (CSS) analysis only involved deaths caused by PSC.. The continuous variable of age was grouped into 2 groups: < 70 and ≥ 70. Marital status was redefined as married, single (separated, divorced, single, widowed), or unknown. Race was reclassified into three groups: black, white, and others (Asian or Pacific Islander, American Indian/Alaska Native, unknown). Variable laterality included left, right and others (paired site, only one side—side unspecified, bilateral). All cases were restaged according to the 8th edition of the American Joint Committee on Cancer (AJCC) TNM staging system. Radiation was reclassified into two groups: yes and no. In the variable of chemotherapy, “yes” referred to all stages of chemotherapy, including concurrent or consecutive chemoradiotherapy, adjuvant chemotherapy, and chemotherapy as the only treatment.

### Statistical analysis

The chi-square test was performed to compare the clinicopathological characteristics between the two groups. The Kaplan‒Meier method was used to calculate overall survival (OS) and cancer-specific survival (CSS). Univariate and multivariate analyses of prognostic factors were performed using Cox proportional hazard regression models. *P* values less than 0.1 in the univariate analysis were included in the multivariate analysis. *P* values less than 0.05 were considered to be statistically significant.

All statistical procedures were performed using R software (R Foundation, Austria) version 4.2.0. The "matchit" package was used for propensity score matching (PSM) analysis and plotting the distribution of the propensity score. The "survival" and "survminer" packages were used to plot survival curves and forest plots.

## Results

### Baseline characteristics

A total of 960 patients with advanced PSC were included in this study. The baseline characteristics are shown in Table 1. Among all the included cases, patients older than 70 years accounted for the majority (54.0%), and male patients accounted for 59.7%. This indicates that PSC predominates in elderly male patients. A majority of the patients were white (80.6%) and married (54.2%). Among the five pathological types of PSC, pleomorphic carcinoma accounted for the most cases (approximately 35.6%), and pulmonary blastoma accounted for only two cases (0.21%). In addition, PSC was mostly located in the right lung (56.1%), and most patients had stage IV disease (53.4%). Of the patients included in the study, 32.6% were treated with surgery, 37.6% with radiotherapy and 45.9% with chemotherapy. The 1-year, 3-year and 5-year OS of all patients were 0.316, 0.189 and 0.157, respectively. The median OS was 6 months and the 95% confidence interval (CI) was 5.251–6.749. The 1-year, 3-year and 5-year CSS were 0.364, 0.234 and 0.215, respectively, and the median CSS was 7 months (95%CI: 6.173–7.827). All patients were divided into two groups according to whether they were treated with chemotherapy. The chi-square test suggested statistically significant differences between the two groups in age, histology, stage, N stage, M stage, surgery, and radiation (*P* < 0.05).

**Table 1:** Comparison of clinicopathological characteristics between original and matched data set

Variables	Original data set				Matched data set			
	**Total**	**Chemotherapy**		***P* value**	**Total**	**Chemotherapy**		***P* value**
		**No/unknown (*N* = 519)**	**Yes (*N* = 441)**			**No (*N* = 321)**	**Yes (*N* = 321)**	
**Age:**				< 0.001				0.937
**< 70**	442 (46.0%)	182 (35.1%)	260 (59.0%)		312 (48.6%)	155 (48.3%)	157 (48.9%)	
**≥ 70**	518 (54.0%)	337 (64.9%)	181 (41.0%)		330 (51.4%)	166 (51.7%)	164 (51.1%)	
**Sex:**				0.719				0.330
**Female**	387 (40.3%)	206 (39.7%)	181 (41.0%)		247 (38.5%)	117 (36.4%)	130 (40.5%)	
**Male**	573 (59.7%)	313 (60.3%)	260 (59.0%)		395 (61.5%)	204 (63.6%)	191 (59.5%)	
**Maritalstatus:**				0.098				0.879
**Married**	520 (54.2%)	265 (51.1%)	255 (57.8%)		353 (55.0%)	179 (55.8%)	174 (54.2%)	
**Single**	401 (41.8%)	233 (44.9%)	168 (38.1%)		263 (41.0%)	130 (40.5%)	133 (41.4%)	
**Unknown**	39 (4.06%)	21 (4.05%)	18 (4.08%)		26 (4.05%)	12 (3.74%)	14 (4.36%)	
**Race:**				0.871				0.985
**Black**	124 (12.9%)	69 (13.3%)	55 (12.5%)		84 (13.1%)	42 (13.1%)	42 (13.1%)	
**Others**	62 (6.46%)	32 (6.17%)	30 (6.80%)		35 (5.45%)	18 (5.61%)	17 (5.30%)	
**White**	774 (80.6%)	418 (80.5%)	356 (80.7%)		523 (81.5%)	261 (81.3%)	262 (81.6%)	
**Laterality:**				0.962				0.804
**Left**	407 (42.4%)	221 (42.6%)	186 (42.2%)		278 (43.3%)	140 (43.6%)	138 (43.0%)	
**Others**	14 (1.46%)	8 (1.54%)	6 (1.36%)		6 (0.93%)	2 (0.62%)	4 (1.25%)	
**Right**	539 (56.1%)	290 (55.9%)	249 (56.5%)		358 (55.8%)	179 (55.8%)	179 (55.8%)	
**Histology:**				0.020				0.918
**Carcinosarcoma**	172 (17.9%)	90 (17.3%)	82 (18.6%)		125 (19.5%)	60 (18.7%)	65 (20.2%)	
**Giant cell carcinoma**	144 (15.0%)	79 (15.2%)	65 (14.7%)		103 (16.0%)	50 (15.6%)	53 (16.5%)	
**Pleomorphic carcinoma**	342 (35.6%)	168 (32.4%)	174 (39.5%)		232 (36.1%)	117 (36.4%)	115 (35.8%)	
**Pulmonary blastoma**	2 (0.21%)	0 (0.00%)	2 (0.45%)		0 (0%)	0 (%)	0 (0%)	
**Spindle cell carcinoma**	300 (31.2%)	182 (35.1%)	118 (26.8%)		182 (28.3%)	94 (29.3%)	88 (27.4%)	
**Stage:**				0.002				0.991
**II**	326 (34.0%)	166 (32.0%)	160 (36.3%)		215 (33.5%)	107 (33.3%)	108 (33.6%)	
**III**	121 (12.6%)	51 (9.83%)	70 (15.9%)		71 (11.1%)	36 (11.2%)	35 (10.9%)	
**IV**	513 (53.4%)	302 (58.2%)	211 (47.8%)		356 (55.5%)	178 (55.5%)	178 (55.5%)	
**TStage:**				0.385				0.935
**T0**	9 (0.94%)	4 (0.77%)	5 (1.13%)		5 (0.78%)	2 (0.62%)	3 (0.93%)	
**T1**	56 (5.83%)	25 (4.82%)	31 (7.03%)		34 (5.30%)	16 (4.98%)	18 (5.61%)	
**T2**	225 (23.4%)	120 (23.1%)	105 (23.8%)		161 (25.1%)	84 (26.2%)	77 (24.0%)	
**T3**	334 (34.8%)	192 (37.0%)	142 (32.2%)		222 (34.6%)	112 (34.9%)	110 (34.3%)	
**T4**	336 (35.0%)	178 (34.3%)	158 (35.8%)		220 (34.3%)	107 (33.3%)	113 (35.2%)	
**NStage:**				< 0.001				0.910
**N0**	390 (40.6%)	244 (47.0%)	146 (33.1%)		250 (38.9%)	124 (38.6%)	126 (39.3%)	
**N1**	144 (15.0%)	63 (12.1%)	81 (18.4%)		104 (16.2%)	53 (16.5%)	51 (15.9%)	
**N2**	333 (34.7%)	166 (32.0%)	167 (37.9%)		221 (34.4%)	113 (35.2%)	108 (33.6%)	
**N3**	93 (9.69%)	46 (8.86%)	47 (10.7%)		67 (10.4%)	31 (9.66%)	36 (11.2%)	
**MStage:**				0.002				1.000
**M0**	447 (46.6%)	217 (41.8%)	230 (52.2%)		286 (44.5%)	143 (44.5%)	143 (44.5%)	
**M1**	513 (53.4%)	302 (58.2%)	211 (47.8%)		356 (55.5%)	178 (55.5%)	178 (55.5%)	
**Surgery:**				< 0.001				0.934
**No**	647 (67.4%)	378 (72.8%)	269 (61.0%)		424 (66.0%)	213 (66.4%)	211 (65.7%)	
**Yes**	313 (32.6%)	141 (27.2%)	172 (39.0%)		218 (34.0%)	108 (33.6%)	110 (34.3%)	
**Radiation:**				< 0.001				0.935
**No**	599 (62.4%)	369 (71.1%)	230 (52.2%)		392 (61.1%)	197 (61.4%)	195 (60.7%)	
**Yes**	361 (37.6%)	150 (28.9%)	211 (47.8%)		250 (38.9%)	124 (38.6%)	126 (39.3%)	
**Chemotherapy:**				< 0.001				< 0.001
**No/unknown**	519 (54.1%)	519 (100%)	0 (0.00%)		321 (50.0%)	321 (100%)	0 (0.00%)	
**Yes**	441 (45.9%)	0 (0.00%)	441 (100%)		321 (50.0%)	0 (0.00%)	321 (100%)	

### Prognostic factors for OS and CSS

**Fig. 2:**
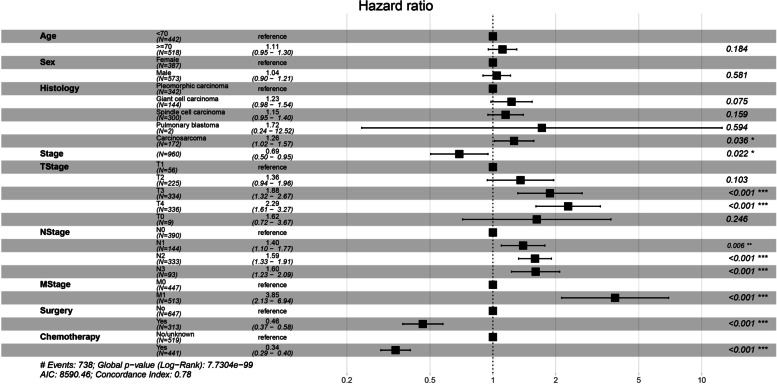
Forest plot showing multivariate analysis for OS

**Fig. 3:**
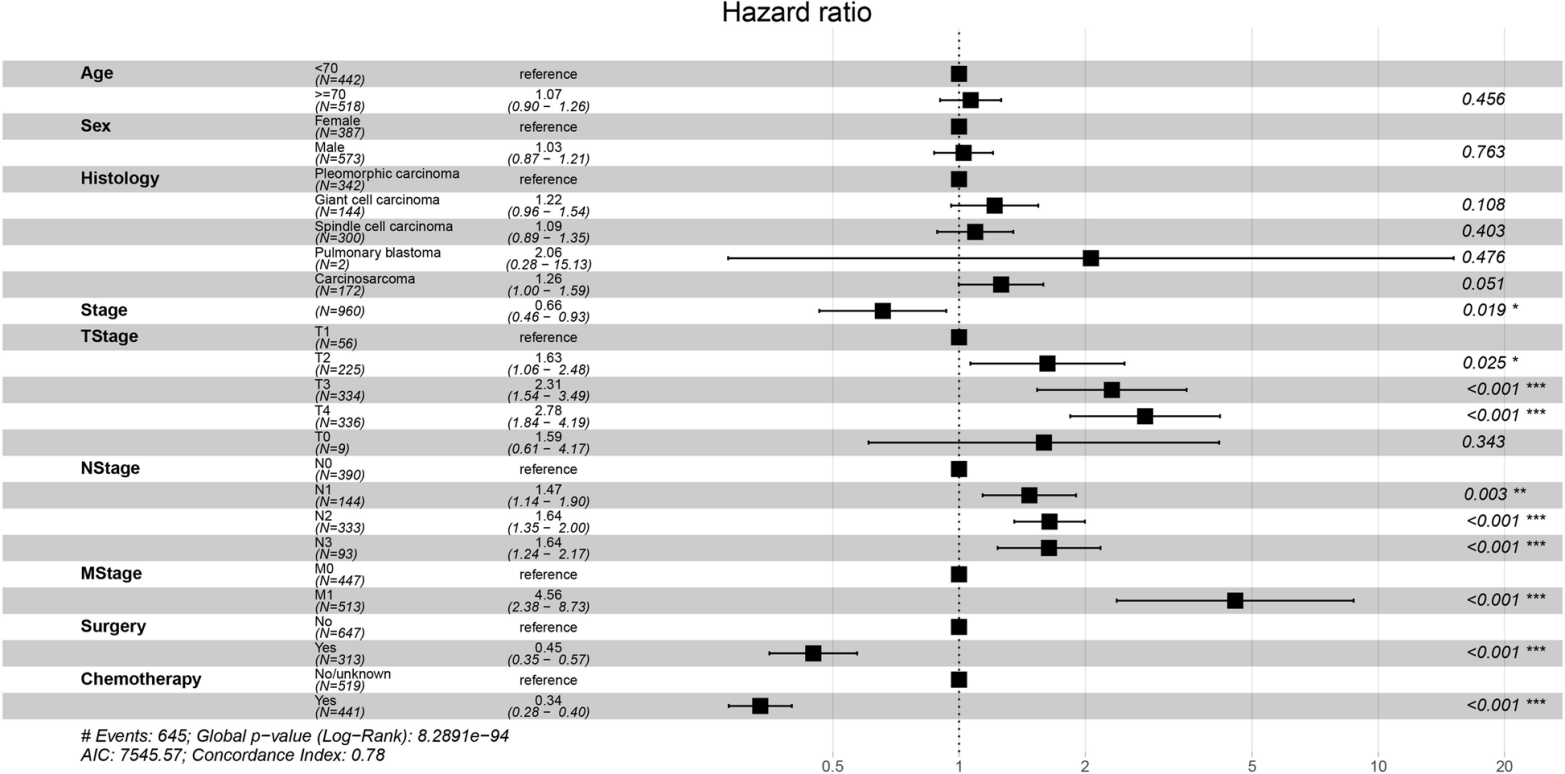
Forest plot showing multivariate analysis for CSS

For all patients included in this study, the Cox proportional hazard model was applied for prognostic analysis. Table 2 shows the results of the univariate analysis of OS and CSS. For OS, the P values for age, sex, histology, stage, T stage, N stage, M stage, surgery, and chemotherapy were less than 0.1. The above variables were included in the multivariate analysis and plotted in a forest plot. As shown in Fig. 2, stage, T stage, N stage, M stage, surgery, and chemotherapy were statistically significant prognostic factors of OS for advanced PSC (*P* < 0.05). For CSS, as shown in Table 2, there were nine variables with p values less than 0.1. These variables were included in the multivariate analysis, and the results are shown in Fig. 3. Stage, T stage, N stage, M stage, surgery, and chemotherapy were statistically significant prognostic factors of CSS for advanced PSC (*P* < 0.05). In conclusion, for both OS and CSS, chemotherapy was a statistically significant prognostic factor. Patients treated with chemotherapy had a better prognosis than those not treated with chemotherapy.

**Table 2:** Univariate analysis of overall survival and cancer-specific survival before propensity score matching

Variables	Total	Overall survival		Cancer-specific survival	
		**HR(95% CI)**	***P* value**	**HR(95% CI)**	***P* value**
**Age:**			< 0.001		0.002
**< 70**	442 (46.0%)	Reference		Reference	
**≥ 70**	518 (54.0%)	1.35 (1.17–1.56)		1.29 (1.10–1.50)	
**Sex:**			0.014		0.026
**Female**	387 (40.3%)	Reference		Reference	
**Male**	573 (59.7%)	1.20 (1.03–1.39)		1.19 (1.02–1.40)	
**Maritalstatus:**			0.279		0.161
**Married**	520 (54.2%)	Reference		Reference	
**Single**	401 (41.8%)	1.13 (0.97–1.31)		1.17 (1.00–1.37)	
**Unknown**	39 (4.06%)	0.97 (0.66–1.41)		0.98 (0.65–1.48)	
**Race:**			0.393		0.334
**Black**	124 (12.9%)	Reference		Reference	
**Others**	62 (6.46%)	0.94 (0.66–1.33)		1.02 (0.71–1.47)	
**White**	774 (80.6%)	0.87 (0.70–1.07)		0.87 (0.69–1.09)	
**Laterality:**			0.575		0.771
**Left**	407 (42.4%)	Reference		Reference	
**Others**	14 (1.46%)	1.34 (0.74–2.45)		1.28 (0.66–2.49)	
**Right**	539 (56.1%)	0.97 (0.84–1.13)		1.01 (0.86–1.18)	
**Histology:**			< 0.001		< 0.001
**Carcinosarcoma**	172 (17.9%)	Reference		Reference	
**Giant cell carcinoma**	144 (15.0%)	1.37 (1.08–1.74)		1.39 (1.08–1.80)	
**Pleomorphic carcinoma**	342 (35.6%)	0.73 (0.59–0.91)		0.73 (0.58–0.92)	
**Pulmonary blastoma**	2 (0.21%)	0.54 (0.08–3.89)		0.60 (0.08–4.32)	
**Spindle cell carcinoma**	300 (31.2%)	1.28 (1.04–1.58)		1.23 (0.98–1.53)	
**Stage:**			< 0.001		< 0.001
**II**	326 (34.0%)	Reference		Reference	
**III**	121 (12.6%)	0.87 (0.64–1.17)		0.84 (0.60–1.17)	
**IV**	513 (53.4%)	2.78 (2.35–3.28)		3.02 (2.53–3.62)	
**TStage:**			< 0.001		< 0.001
**T0**	9 (0.94%)	1.90 (0.84–4.26)		1.88 (0.72–4.90)	
**T1**	56 (5.83%)	Reference		Reference	
**T2**	225 (23.4%)	1.36 (0.95–1.95)		1.64 (1.08–2.49)	
**T3**	334 (34.8%)	1.69 (1.19–2.39)		2.05 (1.37–3.08)	
**T4**	336 (35.0%)	2.12 (1.49–3.00)		2.58 (1.72–3.87)	
**NStage:**			< 0.001		< 0.001
**N0**	390 (40.6%)	Reference		Reference	
**N1**	144 (15.0%)	0.86 (0.68–1.08)		0.90 (0.70–1.15)	
**N2**	333 (34.7%)	1.53 (1.30–1.81)		1.61 (1.35–1.93)	
**N3**	93 (9.69%)	1.91 (1.49–2.44)		1.98 (1.52–2.57)	
**MStage:**			< 0.001		< 0.001
**M0**	447 (46.6%)	Reference		Reference	
**M1**	513 (53.4%)	2.87 (2.46–3.34)		3.14 (2.66–3.71)	
**Surgery:**			< 0.001		< 0.001
**No**	647 (67.4%)	Reference		Reference	
**Yes**	313 (32.6%)	0.29 (0.24–0.34)		0.27 (0.22–0.33)	
**Radiation:**			0.237		0.876
**No**	599 (62.4%)	Reference		Reference	
**Yes**	361 (37.6%)	0.91 (0.78–1.05)		0.98 (0.84–1.15)	
**Chemotherapy:**			< 0.001		< 0.001
**No/unknown**	519 (54.1%)	Reference		Reference	
**Yes**	441 (45.9%)	0.40 (0.34–0.46)		0.40 (0.34–0.47)

### Survival analysis after PSM

**Fig. 4:**
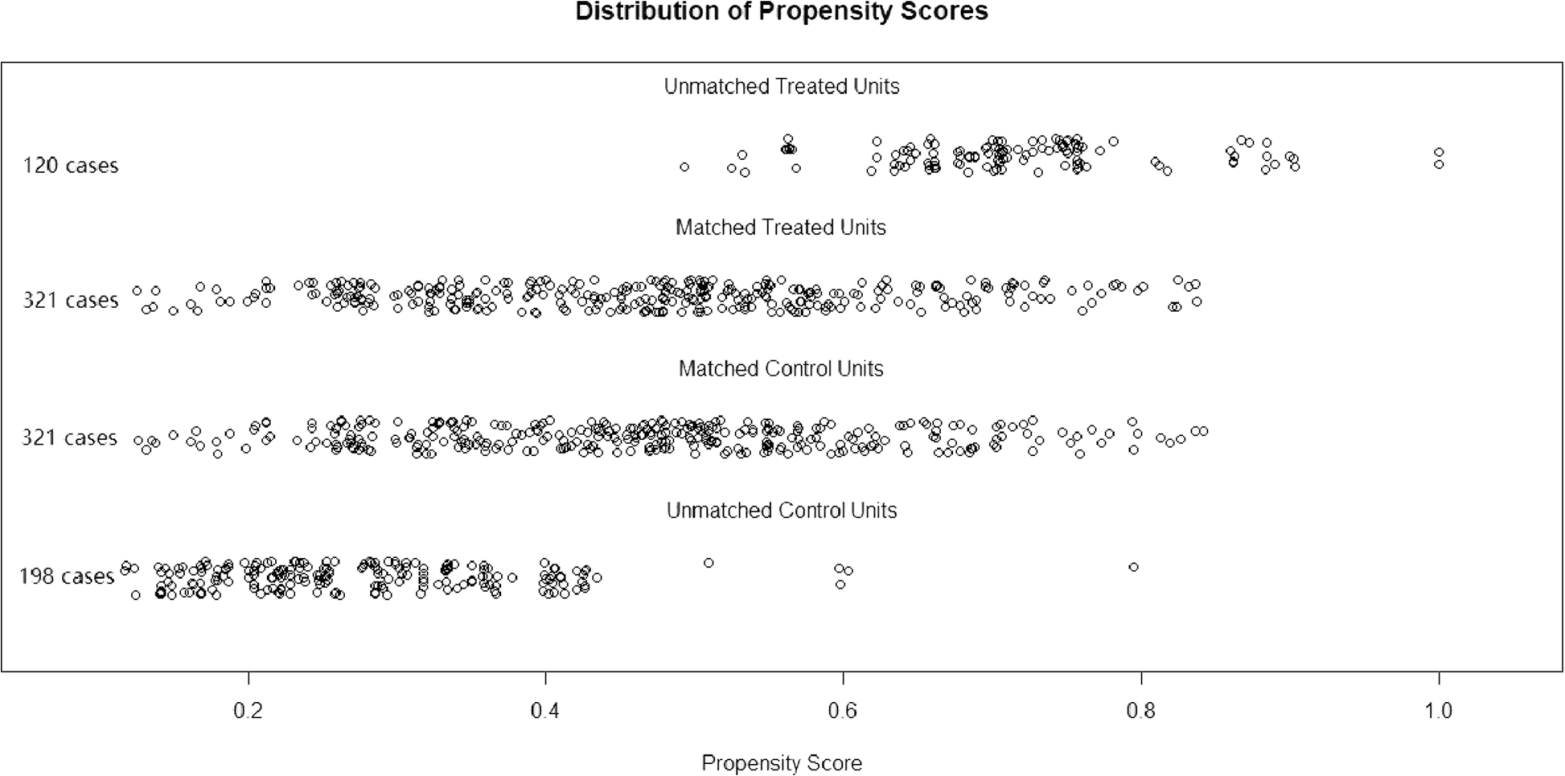
Distribution of propensity score

**Fig. 5:**
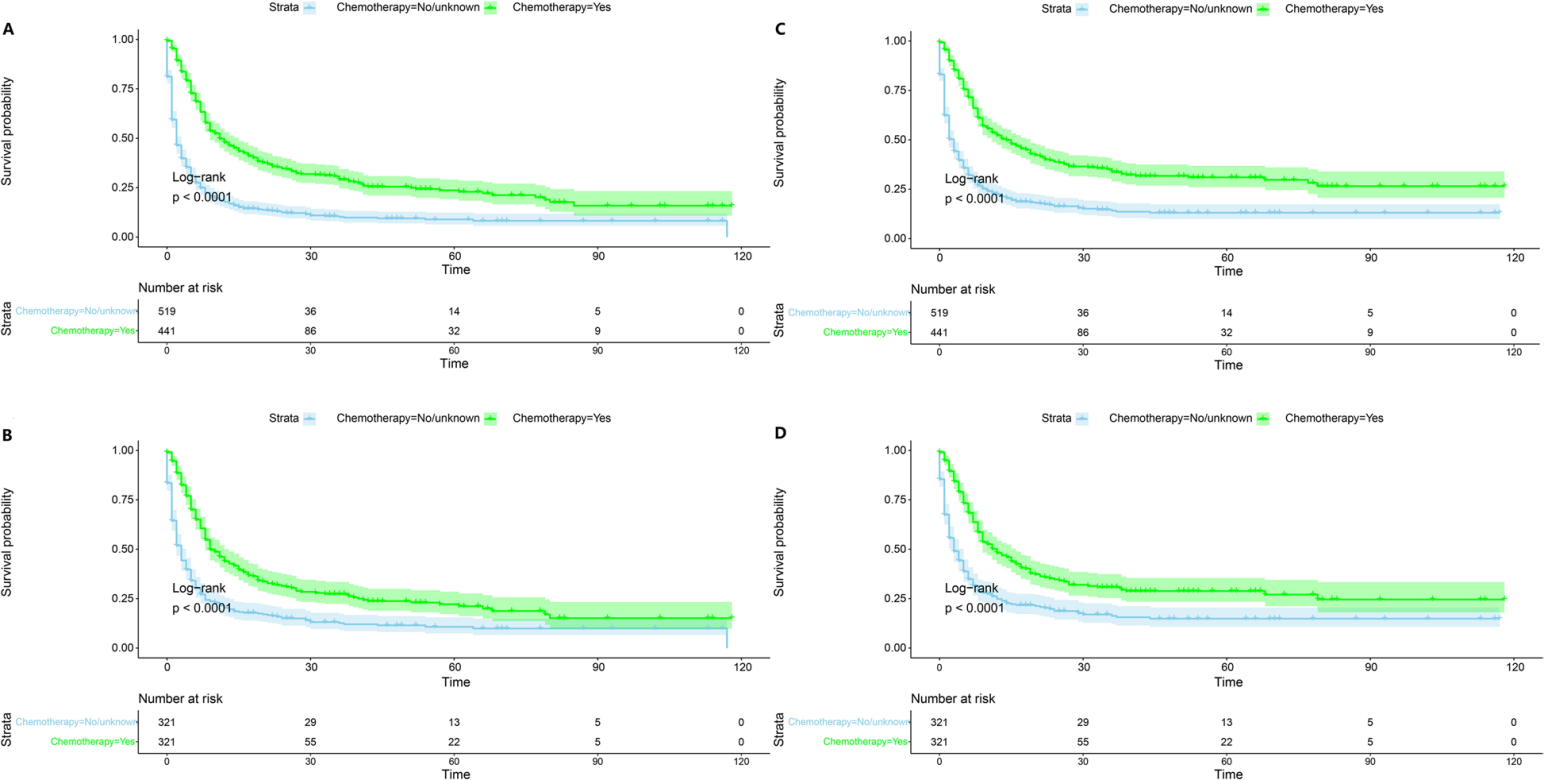
Kaplan–Meier curves of Overall Survival (**A**,**C**) and Cancer-specific Survival (**B**,**D**) before and after propensity score matching

As shown in Table 1, we divided all patients into a chemotherapy group and a no-chemotherapy group. In the original data set, significant differences (*P* < 0.05) were found between the two groups in the variables age, histology, stage, N stage, M stage, surgery, and radiation. To reduce potential bias, we performed 1-to-1 PSM for all variables, and the distribution of propensity scores is shown in Fig. 4. As shown in the matched data set in Table 1, a total of 642 cases were obtained after PSM, with no significant difference between the two groups for all variables. Kaplan‒Meier curves were plotted separately for the original data set matched data set, as shown in Fig. 5. All *P* values were less than 0.05 both before and after PSM, which also indicates that for OS and CSS, the prognosis was significantly better in the chemotherapy group than in the no-chemotherapy group.

## Discussion

This study extracted PSC cases from the SEER database for the first time to study the therapeutic significance of chemotherapy for advanced PSC and included a total of 960 patients with stage II-IV disease. Compared to previous studies on PSC, the number of cases was greatly increased. Since chemotherapy is not recommended for stage I NSCLC according to current NSCLC guidelines, patients with stage I were not included in this study. Survival analysis of the enrolled cases and exploration of prognostic factors for PSC using the Cox hazard proportional model revealed that chemotherapy was a statistically significant prognostic factor for both OS and CSS (*P* < 0.001) (Figs. 2 and 3). Subsequently, we aimed to reduce potential bias, especially to balance the prognostic impact of TNM stage, age, sex, and treatment modality such as surgery and radiotherapy. Applying propensity score matching, patients were divided into two groups according to chemotherapy in a 1-to-1 ratio, and by plotting survival curves and comparing OS and CSS between the two groups, we found that OS and CSS were significantly better in the chemotherapy group than in the non-chemotherapy group (*P* < 0.001) (Fig. 5). We finally concluded that for advanced PSC, chemotherapy significantly enhanced the OS and CSS of patients.

Several studies have been conducted to determine the efficacy of chemotherapy in the treatment of advanced PSC. Some studies revealed its resistance to chemotherapy, while others addressed the benefits of chemotherapy. Hendricksen et al. reported a study of 310 patients with PSC that found no significant difference in 5-year OS between patients treated with postoperative chemotherapy and those not treated with chemotherapy (*P* = 0.2868) [20]. A study by Lococo et al. that included 142 patients with PSC confirmed that adjuvant chemotherapy did not show a significant benefit in 5-year OS and long-term survival (LTS) (*P* = 0.293) [21]. Karim et al. investigated 25 patients with PSC. They found that chemotherapy did not result in a significant survival improvement in patients with stage III and IV disease (*P* = 0.451) [22]. Conversely, there are some studies suggesting that chemotherapy can benefit patients with PSC. Huang et al. reported a study of 51 patients with PSC that found a significant increase in 5-year OS in patients who were treated with chemotherapy compared to those who were not (38.4% vs. 8.5%, *p* = 0.029) [14]. A study by Sun et al. suggested that in younger patients with advanced PSC, chemotherapy may yield an improved prognosis [23]. Hou et al. evaluated 114 patients with PSC and found that postoperative chemotherapy could achieve a significant survival benefit [24]. The above studies on the effect of chemotherapy on the treatment of PSC have drawn different conclusions. Most of these studies focused on specific PSC patients. For example, the studies of Hendricksen, Lococo and Hou are mainly for patients receiving adjuvant chemotherapy. Sun's study focused on younger PSC patients. In this study, all eligible advanced PSCs in the SEER database were included in my research, the purpose is to explore the therapeutic effect of chemotherapy on advanced PSC patients. In addition, the sample sizes of the above studies are all small. For example, the sample sizes of Karim and Huang's studies are less than 100 cases, and even the study of Hendricksen with the largest sample size is only 310 cases. Small sample sizes are likely to cause bias. The sample size of this study is 960 cases, which is significantly larger than the sample size of the above studies. Therefore, the conclusions of this study are more valuable.

Although this study confirmed that chemotherapy has a positive effect on improving the prognosis of advanced PSC, information on the chemotherapy regimen for patients is not known because it is not available in the SEER database. Chemotherapy regimens for PSC currently follow the guidelines for NSCLC, but there is controversy about the therapeutic effects of different chemotherapy regimens. Some retrospective studies have shown that PSC is not sensitive to many chemotherapy regimens [25, 26]. However, other studies have shown that platinum-based chemotherapy regimens can significantly improve OS in patients with PSC [19, 27]. In addition, some studies have shown that gemcitabine plus docetaxel regimens are superior to platinum-based regimens [28]. Therefore, more prospective multicenter studies are needed in the future to explore the most appropriate chemotherapy regimen for PSC.

Since the data for this study were extracted from the SEER database, there are some limitations. First, this study is a retrospective study, and selection bias is inevitable. However, this study still minimized bias through multivariate regression analysis as well as PSM. Second, the impact of different chemotherapy regimens on treatment could not be assessed because specific chemotherapy regimens were not available in the SEER database, at the same time, the data in the SEER database did not indicate whether radiotherapy was palliative or curative, which would also affect the results of survival analysis. Additionally, there is no information such as patient physical status or comorbid diseases that would potentially affect prognosis. Finally, due to the low incidence of PSC, no external data were collected in this study to further validate the findings .

## Conclusions

For advanced PSC, chemotherapy can significantly improve the OS and CSS of patients. Chemotherapy should be an important part of PSC treatment.

## Data Availability

The raw data supporting the conclusions of this article will be made available by the authors, without undue reservation (https://figshare.com/articles/dataset/PSCchemo_sav/22178606). The download address of Seer*stat software is https://seer.cancer.gov/seerstat/download/. The detailed database information is Surveillance, Epidemiology, and End Results (SEER) Program (www.seer.cancer.gov) SEER*Stat Database: Incidence—SEER Research Plus Data, 17 Registries, Nov 2021 Sub (1975–2020)—Linked To County Attributes—Time Dependent (1990–2020) Income/Rurality, 1969–2020 Counties, National Cancer Institute, DCCPS, Surveillance Research Program, released April 2022, based on the November 2021 submission.

## Abbreviations

NSCLC: Non-small cell lung cancer
PSM: Propensity score matching
PSC: Pulmonary sarcomatoid carcinoma
SEER: Surveillance, Epidemiology, and End Results
CSS: Cancer-specific survival
OS: Overall survival
AJCC: American Joint Committee on Cancer
CI: Confidence interval
DCCPS: Division of Cancer Control and Population Sciences
